# Development and optimization of PSCA-specific CAR T cells for the treatment of bone metastatic prostate cancer

**DOI:** 10.1186/2051-1426-3-S2-P115

**Published:** 2015-11-04

**Authors:** Ethan Gerdts, Saul Priceman, Dileshni Tilakawardane, Anthony Park, Wen-Chung Chang, Sarah Wright, Christine E Brown, Stephen J Forman

**Affiliations:** 1 City of Hope National Medical Center, Duarte, CA, USA; 2 Beckman Research Institute, City of Hope National Medical Center, Duarte, CA, USA

## 

Prostate Cancer (PCa) is the third most common cancer type in the United States, with over 200,000 new cases projected to be diagnosed this year. In approximately 80% of PCa patients, tumor phenotype includes overexpression of prostate stem cell antigen, or PSCA. Furthermore, PSCA is expressed on nearly 100% of bone metastatic prostate cancers, making it an attractive immunotherapeutic target. We have genetically engineered T cells to express chimeric antigen receptors (CARs) which specifically target PSCA. Recent clinical trials with CARs targeting CD19 for B-cell malignancies have demonstrated impressive results, yet replicating this success with other antigen targets remains elusive. Immunotherapy against solid tumors poses a more difficult tumor challenge because of the immunosuppressive microenvironment that can significantly hinder CAR efficacy. Additionally, there have been instances of on-target, off-tumor toxicity due to low levels of antigen expression on normal tissue.

In the current project we have modified various components of our CAR constructs to improve specificity and overall therapeutic efficacy. Through various *in vitro* functional assays and *in vivo* xenograft models, we have evaluated and optimized a PSCA-targeting CAR. We have compared two single-chain variable fragments with different paratopes, namely the A11 and the MB1 scFvs. While both show comparable potency, the MB1 scFv exhibits nonspecific activity against PSCA-negative tumor lines. Similarly, our data suggest that the 28ζ-costimulatory domain, regardless of linker length, also shows non-specific activation and killing of PSCA-negative tumor lines as compared to the 4-1BB costimulatory domain. Finally, we have demonstrated differences between long, middle, and short linker lengths in intracellular cytokine production, activation, and killing capacities *in vitro* and *in vivo*. By modifying both the ectodomain and intracellular region, we are able to improve the specificity and functionality of our PSCA-CARs, which is essential to developing effective immunotherapies for this advanced disease.

